# Crystal structure of 6-(4-chloro­phen­yl)-6a-nitro-6,6a,6b,7,9,11a-hexa­hydro­spiro[chromeno[3′,4′:3,4]pyrrolo­[1,2-*c*]thia­zole-11,11′-indeno­[1,2-*b*]quinoxaline] chloro­form monosolvate

**DOI:** 10.1107/S1600536814020601

**Published:** 2014-09-20

**Authors:** Nithya Sivakumar, Vijayan Viswanathan, Jonnalagadda Naga Siva Rao, Raghavachary Raghunathan, Devadasan Velmurugan

**Affiliations:** a Mission San Jose High School, Palm AVE, Fremont, CA 94539, USA; b Centre of Advanced Study in Crystallography and Biophysics, University of Madras, Guindy Campus, Chennai 600 025, India; c Department of Organic Chemistry, University of Madras, Guindy Campus, Chennai 600 025, India

**Keywords:** crystal structure, thia­zole, C—H⋯N hydrogen bonding

## Abstract

In the title compound, C_33_H_23_ClN_4_O_3_S·CHCl_3_, the thia­zole ring adopts an envelope conformation with the N atom as the flap, and the pyrrolidine ring adopts a half-chair conformation. The thia­zole ring mean plane makes a dihedral angle of 59.31 (1)° with the pyrrolidine ring mean plane, 71.67 (1)° with the chromene ring and 82.59 (1)° with the chloro­benzene ring. An intra­molecular C—H⋯N hydrogen bond occurs. In the crystal, a second C—H⋯N hydrogen bond links the main and solvent mol­ecules. The solvent chloroform molecule is disordered about two positions with an occupancy ratio of 0.508 (14):0.492 (14).

## Related literature   

For the biological activity of thia­zole derivatives, see: Shao *et al.* (2004 ▶); Hökelek *et al.* (2006 ▶); Muralikrishna *et al.*(2013 ▶); Shruthy & Shakkeela (2014 ▶). 
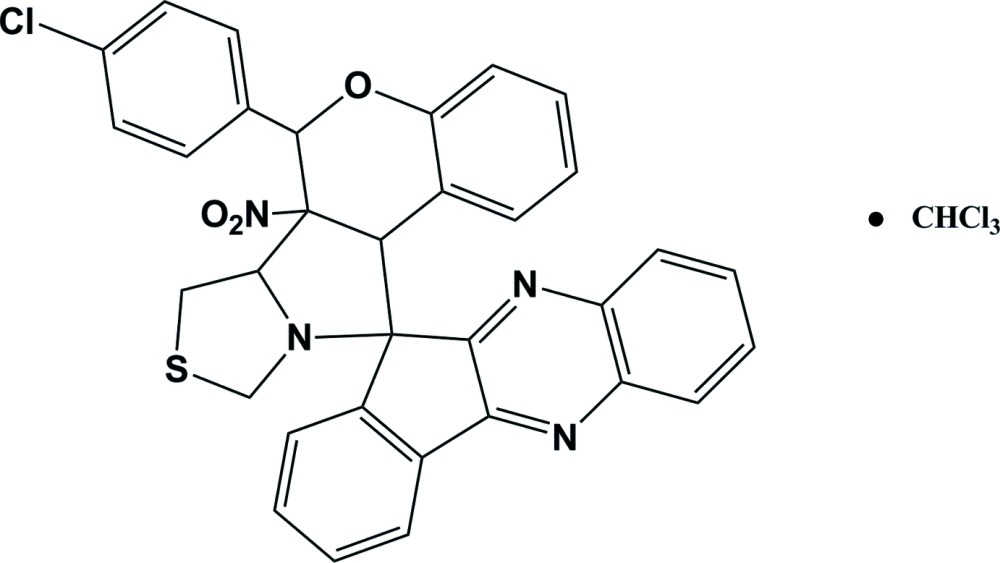


## Experimental   

### Crystal data  

C_33_H_23_ClN_4_O_3_S·CHCl_3_
*M* *_r_* = 710.43
Triclinic, 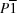

*a* = 8.983 (5) Å
*b* = 13.241 (5) Å
*c* = 14.269 (5) Å
α = 99.890 (5)°
β = 99.204 (5)°
γ = 105.519 (5)°
*V* = 1572.7 (12) Å^3^
*Z* = 2
Mo *K*α radiation
μ = 0.49 mm^−1^
*T* = 293 K
0.20 × 0.15 × 0.10 mm

### Data collection  

Bruker SMART APEXII area-detector diffractometer
Absorption correction: multi-scan (*SADABS*; Bruker, 2008 ▶) *T*_min_ = 0.909, *T*_max_ = 0.953
24128 measured reflections
6440 independent reflections
5331 reflections with *I* > 2σ(*I*)
*R*_int_ = 0.027

### Refinement  

*R*[*F* ^2^ > 2σ(*F* ^2^)] = 0.043
*wR*(*F* ^2^) = 0.121
*S* = 1.04
6440 reflections
443 parameters
10 restraints
H atoms treated by a mixture of independent and constrained refinement
Δρ_max_ = 0.26 e Å^−3^
Δρ_min_ = −0.35 e Å^−3^

### 

Data collection: *APEX2* (Bruker, 2008 ▶); cell refinement: *SAINT* (Bruker, 2008 ▶); data reduction: *SAINT*; program(s) used to solve structure: *SHELXS97* (Sheldrick, 2008 ▶); program(s) used to refine structure: *SHELXL97* (Sheldrick, 2008 ▶); molecular graphics: *ORTEP-3 for Windows* (Farrugia, 2012 ▶); software used to prepare material for publication: *SHELXL97* and *PLATON* (Spek, 2009 ▶).

## Supplementary Material

Crystal structure: contains datablock(s) global, I. DOI: 10.1107/S1600536814020601/rn2127sup1.cif

Structure factors: contains datablock(s) I. DOI: 10.1107/S1600536814020601/rn2127Isup2.hkl

**Fig. 1.**
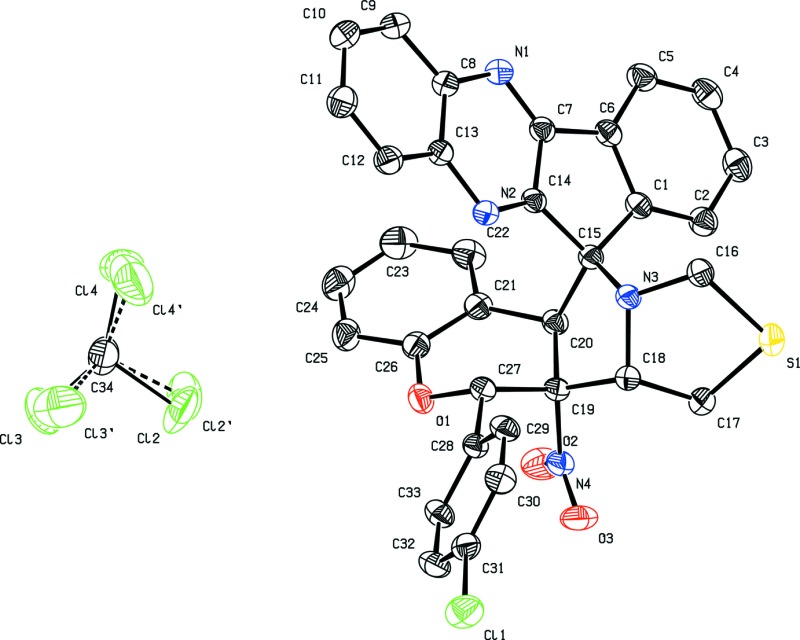
The molecular structure of the title compound, showing the atomic numbering and displacement ellipsoids drawn at 30% probability level. For the sake of clarity, the solvent molecule CHCl3 is omitted.

3 . DOI: 10.1107/S1600536814020601/rn2127fig1.tif
The mol­ecular structure of the title compound, showing the atomic numbering and displacement ellipsoids drawn at 30% probability level. For the sake of clarity, the solvent mol­ecule CHCl_3_ is omitted.

**Fig. 2.**
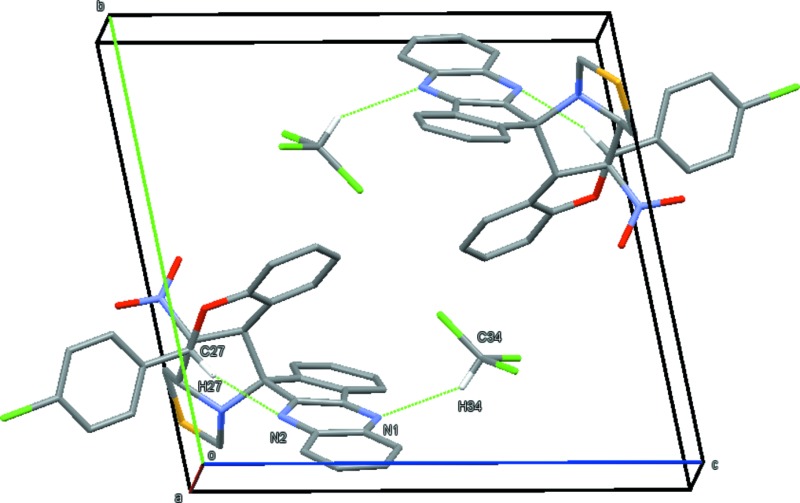
The crystal packing of the title compound viewed down the 'a' axis. H-atoms not involved in H-bonds have been excluded for clarity.

. DOI: 10.1107/S1600536814020601/rn2127fig2.tif
The crystal packing of the title compound viewed down the ’a′ axis. H-atoms not involved in H-bonds have been excluded for clarity.

CCDC reference: 1024312

Additional supporting information:  crystallographic information; 3D view; checkCIF report

## Figures and Tables

**Table 1:** Hydrogen-bond geometry (Å, °)

*D*—H⋯*A*	*D*—H	H⋯*A*	*D*⋯*A*	*D*—H⋯*A*
C34—H34⋯N1	0.98	2.59	3.401 (3)	140
C27—H27⋯N2	0.98	2.27	3.200 (3)	158

## supplementary crystallographic information

### S1. Comment

Thiazole and its derivatives play an important role in medicinal chemistry as herbicidal, fungicidal, bacterial, antitumor agents (Muralikrishna *et al.*,2013). Thiazole derivatives have a wide range of pharmacological applications, such as anticancer, antiviral, antibacterial, antifungal, and anti-inflammatory activities (Shruthy *et al.*,2014). Thiazoles and their annelated derivatives are reported to exhibit diverse biological activities as antituberculous, bacteriostatic and fungistatic agents (Shao *et al.*, 2004). The bioactivity of S,N-thiazoles is mainly due to their structural similarities with protein imidazolyl entities as well as their biological, structural, electronic and spectroscopic properties (Hökelek *et al.*, 2006)

The ORTEP plot of the molecule is shown in Fig. 1. In the title compound, C_33_H_23_N_4_O_3_S_1_Cl_1_.CHCl_3_, the thiazole ring adopts an envelope conformation and pyrrolidine ring adopts a half chair conformation and quinoxaline ring adopts a planar conformation. The thiazole ring makes a dihedral angle of 59.31 (1) ° with the pyrrolidine ring (C15-N3-C18-C19-C20), 71.67 (1) ° with the chromene ring (C19-C20-C21-C22-C23-C24-C25-C26-O1-C27) and 82.59 (1) ° with the chlorobenzene (C28-C29-C30-C31-C32-C33-CL1). The pyrrolidine ring makes a dihedral angle of 51.98 (1) ° with the chromene ring and 23.49 (1) ° with the chlorobenzene ring. The nitro group makes a dihedral angle of 27.93 (3) ° with the pyrrolidine ring. In the chlorobenzene molecule, the chlorine atom deviates by 0.050 (1)Å from the benzene ring.

In the crystal, the molecular structure has an intramolecular C—H···N hydrogen bond. The crystal packing of the title compound viewed down the 'a' axis is shown in Fig. 2. The solvent molecule (CHCl_3_) has an intermolecular C—H···N hydrogen bond (Table. 1.).

### S2. Experimental

To a solution of indenoquinoxalinone (1.0 mmol) and thiazolidine-4-carboxylic acid (1.5 mmol) in dry toluene, was added 2-(4-chlorophenyl)-3-nitro- 2H-chromene (1 mmol) under nitrogen atmosphere. The solution was refluxed for 20 h in Dean-Stark apparatus to give the corresponding cycloadduct. After completion of the reaction as indicated by TLC, the solvent was evaporated under reduced pressure.The crude product obtained was purifed by column chromatography using hexane/EtOAc (8:2) as eluent (Yield: 87%).

### S3. Refinement

The hydrogen atoms were placed in calculated positions with C—H = 0.93Å to 0.98Å, refined in the riding model with fixed isotropic displacement parameters:*U*_iso_(H) = 1.5*U*_eq_(C) for methyl group and *U*_iso_(H) = 1.2*U*_eq_(C) for other groups. In solvent molecules the chlorine atoms Cl2, Cl3 & Cl4 are disordered over two orientations 0.508 (14) : 0.492 (14). The bond distance between the carbon and chlorine atom was restrained to 1.782 (1) Å.

### Crystal data

**Table:**

C_33_H_23_ClN_4_O_3_S·CHCl_3_	*Z* = 2
*M**_r_* = 710.43	*F*(000) = 728
Triclinic, *P*1	*D*_x_ = 1.500 Mg m^−^^3^
Hall symbol: -P 1	Mo *K*α radiation, λ = 0.71073 Å
*a* = 8.983 (5) Å	Cell parameters from 6441 reflections
*b* = 13.241 (5) Å	θ = 1.5–26.5°
*c* = 14.269 (5) Å	µ = 0.49 mm^−^^1^
α = 99.890 (5)°	*T* = 293 K
β = 99.204 (5)°	Block, colourless
γ = 105.519 (5)°	0.20 × 0.15 × 0.10 mm
*V* = 1572.7 (12) Å^3^	

### Data collection

**Table:**

Bruker SMART APEXII area-detector diffractometer	6440 independent reflections
Radiation source: fine-focus sealed tube	5331 reflections with *I* > 2σ(*I*)
Graphite monochromator	*R*_int_ = 0.027
ω and φ scans	θ_max_ = 26.5°, θ_min_ = 1.5°
Absorption correction: multi-scan (*SADABS*; Bruker, 2008)	*h* = −11→10
*T*_min_ = 0.909, *T*_max_ = 0.953	*k* = −16→16
24128 measured reflections	*l* = −17→17

### Refinement

**Table:**

Refinement on *F*^2^	Primary atom site location: structure-invariant direct methods
Least-squares matrix: full	Secondary atom site location: difference Fourier map
*R*[*F*^2^ > 2σ(*F*^2^)] = 0.043	Hydrogen site location: inferred from neighbouring sites
*wR*(*F*^2^) = 0.121	H atoms treated by a mixture of independent and constrained refinement
*S* = 1.04	*w* = 1/[σ^2^(*F*_o_^2^) + (0.0547*P*)^2^ + 0.7757*P*] where *P* = (*F*_o_^2^ + 2*F*_c_^2^)/3
6440 reflections	(Δ/σ)_max_ < 0.001
443 parameters	Δρ_max_ = 0.26 e Å^−^^3^
10 restraints	Δρ_min_ = −0.35 e Å^−^^3^

### Special details

**Table:**

Geometry. All esds (except the esd in the dihedral angle between two l.s. planes) are estimated using the full covariance matrix. The cell esds are taken into account individually in the estimation of esds in distances, angles and torsion angles; correlations between esds in cell parameters are only used when they are defined by crystal symmetry. An approximate (isotropic) treatment of cell esds is used for estimating esds involving l.s. planes.
Refinement. Refinement of F^2^ against ALL reflections. The weighted R-factor wR and goodness of fit S are based on F^2^, conventional R-factors R are based on F, with F set to zero for negative F^2^. The threshold expression of F^2^ > 2sigma(F^2^) is used only for calculating R-factors(gt) etc. and is not relevant to the choice of reflections for refinement. R-factors based on F^2^ are statistically about twice as large as those based on F, and R- factors based on ALL data will be even larger.

### Fractional atomic coordinates and isotropic or equivalent isotropic displacement parameters (Å^2^)

**Table:**

	*x*	*y*	*z*	*U*_iso_*/*U*_eq_	Occ. (<1)
C1	0.9558 (2)	0.24923 (14)	0.26430 (14)	0.0378 (4)	
C2	1.1139 (2)	0.28740 (17)	0.26134 (16)	0.0462 (5)	
H2	1.1429	0.3090	0.2065	0.055*	
C3	1.2286 (3)	0.29292 (18)	0.34150 (17)	0.0514 (5)	
H3	1.3349	0.3168	0.3393	0.062*	
C4	1.1875 (3)	0.26356 (18)	0.42414 (16)	0.0506 (5)	
H4	1.2662	0.2682	0.4771	0.061*	
C5	1.0308 (3)	0.22736 (17)	0.42910 (15)	0.0472 (5)	
H5	1.0029	0.2085	0.4852	0.057*	
C6	0.9154 (2)	0.21967 (15)	0.34866 (14)	0.0392 (4)	
C7	0.7434 (2)	0.17972 (15)	0.33296 (13)	0.0368 (4)	
C8	0.4989 (2)	0.10010 (16)	0.35382 (14)	0.0398 (4)	
C9	0.3958 (3)	0.05880 (19)	0.41235 (16)	0.0507 (5)	
H9	0.4350	0.0647	0.4781	0.061*	
C10	0.2389 (3)	0.01025 (19)	0.37270 (18)	0.0554 (6)	
H10	0.1718	−0.0171	0.4118	0.067*	
C11	0.1773 (3)	0.0009 (2)	0.27431 (18)	0.0559 (6)	
H11	0.0704	−0.0344	0.2481	0.067*	
C12	0.2726 (2)	0.04310 (18)	0.21622 (16)	0.0484 (5)	
H12	0.2302	0.0380	0.1511	0.058*	
C13	0.4351 (2)	0.09437 (15)	0.25498 (14)	0.0380 (4)	
C14	0.6781 (2)	0.18146 (14)	0.23578 (13)	0.0336 (4)	
C15	0.8081 (2)	0.23515 (14)	0.18649 (13)	0.0342 (4)	
C16	0.8824 (3)	0.09580 (17)	0.07376 (16)	0.0453 (5)	
H16A	0.8158	0.0269	0.0332	0.054*	
H16B	0.9282	0.0843	0.1359	0.054*	
C17	0.9659 (3)	0.26781 (19)	−0.00076 (17)	0.0502 (5)	
H17A	1.0285	0.3325	0.0476	0.060*	
H17B	0.9673	0.2819	−0.0652	0.060*	
C18	0.7948 (2)	0.23140 (15)	0.01383 (14)	0.0386 (4)	
H18	0.7234	0.1877	−0.0475	0.046*	
C19	0.7302 (2)	0.32069 (14)	0.05635 (13)	0.0364 (4)	
C20	0.8000 (2)	0.34680 (14)	0.16582 (13)	0.0364 (4)	
H20	0.9086	0.3946	0.1779	0.044*	
C21	0.7101 (2)	0.40258 (14)	0.22642 (14)	0.0402 (4)	
C22	0.7699 (3)	0.44626 (16)	0.32546 (16)	0.0524 (5)	
H22	0.8642	0.4379	0.3556	0.063*	
C23	0.6907 (4)	0.50206 (19)	0.37954 (18)	0.0629 (7)	
H23	0.7307	0.5298	0.4460	0.075*	
C24	0.5537 (3)	0.51652 (19)	0.33561 (19)	0.0623 (7)	
H24	0.5022	0.5554	0.3722	0.075*	
C25	0.4912 (3)	0.47420 (17)	0.23767 (18)	0.0531 (5)	
H25	0.3979	0.4843	0.2080	0.064*	
C26	0.5690 (2)	0.41621 (15)	0.18371 (15)	0.0417 (4)	
C27	0.5473 (2)	0.28777 (15)	0.04070 (14)	0.0380 (4)	
H27	0.5156	0.2305	0.0754	0.046*	
C28	0.4524 (2)	0.24729 (15)	−0.06206 (14)	0.0376 (4)	
C29	0.4170 (3)	0.13901 (16)	−0.10564 (15)	0.0449 (5)	
H29	0.4508	0.0933	−0.0704	0.054*	
C30	0.3326 (3)	0.09856 (17)	−0.20035 (16)	0.0487 (5)	
H30	0.3103	0.0262	−0.2294	0.058*	
C31	0.2816 (2)	0.16635 (18)	−0.25153 (15)	0.0456 (5)	
C32	0.3109 (3)	0.27288 (18)	−0.20948 (16)	0.0502 (5)	
H32	0.2736	0.3175	−0.2444	0.060*	
C33	0.3965 (2)	0.31317 (16)	−0.11469 (16)	0.0452 (5)	
H33	0.4169	0.3854	−0.0859	0.054*	
N1	0.6585 (2)	0.14220 (14)	0.39299 (12)	0.0429 (4)	
N2	0.52868 (19)	0.13826 (12)	0.19513 (11)	0.0370 (3)	
N3	0.79030 (19)	0.16833 (12)	0.08831 (11)	0.0363 (3)	
N4	0.7818 (2)	0.41753 (14)	0.01250 (14)	0.0459 (4)	
O1	0.50296 (17)	0.37741 (11)	0.08576 (10)	0.0462 (3)	
O2	0.8318 (2)	0.50626 (13)	0.06648 (14)	0.0721 (5)	
O3	0.7643 (2)	0.40034 (15)	−0.07547 (12)	0.0647 (5)	
Cl1	0.18217 (8)	0.11507 (6)	−0.37305 (4)	0.0697 (2)	
S1	1.04129 (7)	0.15659 (5)	0.01349 (5)	0.05568 (17)	
C34	0.7767 (3)	0.2814 (2)	0.6293 (2)	0.0704 (7)	
H34	0.7625	0.2172	0.5788	0.084*	
Cl2	0.6187 (6)	0.2425 (5)	0.6852 (5)	0.1032 (16)	0.508 (14)
Cl3	0.9474 (6)	0.2911 (5)	0.7085 (4)	0.0962 (11)	0.508 (14)
Cl4	0.7716 (6)	0.3776 (3)	0.5647 (2)	0.0990 (11)	0.508 (14)
Cl4'	0.7955 (7)	0.4117 (9)	0.6045 (12)	0.168 (3)	0.492 (14)
Cl3'	0.9441 (7)	0.3240 (10)	0.7237 (5)	0.133 (2)	0.492 (14)
Cl2'	0.6026 (7)	0.2434 (3)	0.6676 (6)	0.0987 (15)	0.492 (14)

### Atomic displacement parameters (Å^2^)

**Table:**

	*U*^11^	*U*^22^	*U*^33^	*U*^12^	*U*^13^	*U*^23^
C1	0.0394 (10)	0.0330 (9)	0.0414 (10)	0.0128 (8)	0.0062 (8)	0.0090 (7)
C2	0.0401 (11)	0.0457 (11)	0.0516 (12)	0.0111 (9)	0.0071 (9)	0.0138 (9)
C3	0.0363 (11)	0.0480 (12)	0.0642 (14)	0.0097 (9)	0.0032 (10)	0.0101 (10)
C4	0.0463 (12)	0.0497 (12)	0.0495 (12)	0.0163 (10)	−0.0046 (9)	0.0059 (9)
C5	0.0491 (12)	0.0534 (12)	0.0385 (10)	0.0190 (10)	0.0020 (9)	0.0102 (9)
C6	0.0424 (11)	0.0359 (9)	0.0393 (10)	0.0155 (8)	0.0047 (8)	0.0068 (8)
C7	0.0412 (10)	0.0355 (9)	0.0355 (9)	0.0155 (8)	0.0076 (8)	0.0074 (7)
C8	0.0422 (11)	0.0411 (10)	0.0400 (10)	0.0158 (8)	0.0107 (8)	0.0129 (8)
C9	0.0537 (13)	0.0610 (13)	0.0460 (11)	0.0205 (11)	0.0172 (10)	0.0238 (10)
C10	0.0523 (13)	0.0624 (14)	0.0604 (14)	0.0165 (11)	0.0249 (11)	0.0268 (11)
C11	0.0402 (12)	0.0619 (14)	0.0651 (14)	0.0091 (10)	0.0135 (10)	0.0217 (11)
C12	0.0414 (11)	0.0565 (12)	0.0472 (11)	0.0133 (9)	0.0077 (9)	0.0159 (9)
C13	0.0397 (10)	0.0372 (9)	0.0409 (10)	0.0145 (8)	0.0113 (8)	0.0121 (8)
C14	0.0389 (10)	0.0299 (8)	0.0349 (9)	0.0143 (7)	0.0082 (7)	0.0083 (7)
C15	0.0365 (10)	0.0329 (9)	0.0351 (9)	0.0124 (7)	0.0079 (7)	0.0094 (7)
C16	0.0530 (12)	0.0427 (11)	0.0488 (11)	0.0237 (9)	0.0165 (9)	0.0132 (9)
C17	0.0502 (12)	0.0583 (13)	0.0578 (13)	0.0256 (10)	0.0245 (10)	0.0273 (10)
C18	0.0424 (10)	0.0431 (10)	0.0371 (9)	0.0190 (8)	0.0126 (8)	0.0139 (8)
C19	0.0394 (10)	0.0352 (9)	0.0406 (10)	0.0144 (8)	0.0116 (8)	0.0164 (8)
C20	0.0369 (10)	0.0319 (9)	0.0413 (10)	0.0108 (7)	0.0076 (8)	0.0111 (7)
C21	0.0489 (11)	0.0280 (9)	0.0453 (10)	0.0121 (8)	0.0130 (9)	0.0090 (7)
C22	0.0690 (15)	0.0371 (10)	0.0485 (12)	0.0188 (10)	0.0070 (10)	0.0044 (9)
C23	0.096 (2)	0.0442 (12)	0.0483 (12)	0.0250 (13)	0.0170 (13)	0.0011 (10)
C24	0.0866 (19)	0.0445 (12)	0.0661 (15)	0.0280 (12)	0.0358 (14)	0.0091 (11)
C25	0.0581 (14)	0.0441 (11)	0.0673 (14)	0.0228 (10)	0.0262 (11)	0.0162 (10)
C26	0.0482 (11)	0.0329 (9)	0.0477 (11)	0.0131 (8)	0.0166 (9)	0.0119 (8)
C27	0.0416 (10)	0.0363 (9)	0.0413 (10)	0.0157 (8)	0.0110 (8)	0.0142 (8)
C28	0.0353 (10)	0.0386 (10)	0.0433 (10)	0.0127 (8)	0.0099 (8)	0.0169 (8)
C29	0.0498 (12)	0.0388 (10)	0.0505 (11)	0.0166 (9)	0.0083 (9)	0.0187 (9)
C30	0.0511 (12)	0.0414 (11)	0.0507 (12)	0.0116 (9)	0.0089 (9)	0.0089 (9)
C31	0.0351 (10)	0.0568 (12)	0.0423 (10)	0.0076 (9)	0.0067 (8)	0.0160 (9)
C32	0.0441 (11)	0.0514 (12)	0.0577 (13)	0.0144 (9)	0.0030 (10)	0.0272 (10)
C33	0.0447 (11)	0.0373 (10)	0.0552 (12)	0.0147 (8)	0.0050 (9)	0.0165 (9)
N1	0.0461 (10)	0.0496 (9)	0.0365 (8)	0.0175 (8)	0.0095 (7)	0.0140 (7)
N2	0.0371 (8)	0.0394 (8)	0.0368 (8)	0.0128 (7)	0.0081 (6)	0.0123 (6)
N3	0.0429 (9)	0.0350 (8)	0.0361 (8)	0.0175 (7)	0.0110 (7)	0.0106 (6)
N4	0.0445 (10)	0.0473 (10)	0.0564 (11)	0.0190 (8)	0.0156 (8)	0.0270 (8)
O1	0.0474 (8)	0.0479 (8)	0.0500 (8)	0.0256 (7)	0.0110 (6)	0.0107 (6)
O2	0.0983 (14)	0.0397 (9)	0.0797 (12)	0.0129 (9)	0.0248 (11)	0.0246 (8)
O3	0.0767 (12)	0.0735 (11)	0.0547 (10)	0.0232 (9)	0.0188 (8)	0.0379 (8)
Cl1	0.0578 (4)	0.0930 (5)	0.0459 (3)	0.0096 (3)	−0.0003 (3)	0.0145 (3)
S1	0.0528 (3)	0.0631 (4)	0.0671 (4)	0.0324 (3)	0.0256 (3)	0.0216 (3)
C34	0.0609 (16)	0.0823 (18)	0.0741 (17)	0.0275 (14)	0.0249 (13)	0.0148 (14)
Cl2	0.0616 (18)	0.159 (4)	0.0890 (18)	0.0206 (16)	0.0457 (14)	0.0199 (16)
Cl3	0.0606 (15)	0.104 (2)	0.119 (2)	0.0311 (14)	−0.0016 (12)	0.0197 (15)
Cl4	0.149 (3)	0.0942 (17)	0.080 (2)	0.0599 (16)	0.0426 (13)	0.0360 (13)
Cl4'	0.090 (2)	0.177 (5)	0.299 (9)	0.064 (3)	0.071 (4)	0.157 (6)
Cl3'	0.072 (2)	0.189 (6)	0.121 (3)	−0.001 (3)	−0.0016 (19)	0.072 (4)
Cl2'	0.0620 (17)	0.079 (2)	0.157 (4)	0.0165 (13)	0.037 (2)	0.0274 (19)

### Geometric parameters (Å, º)

**Table:**

C1—C2	1.384 (3)	C18—H18	0.9800
C1—C6	1.400 (3)	C19—N4	1.517 (2)
C1—C15	1.534 (3)	C19—C20	1.529 (3)
C2—C3	1.389 (3)	C19—C27	1.551 (3)
C2—H2	0.9300	C20—C21	1.506 (3)
C3—C4	1.378 (3)	C20—H20	0.9800
C3—H3	0.9300	C21—C26	1.387 (3)
C4—C5	1.378 (3)	C21—C22	1.389 (3)
C4—H4	0.9300	C22—C23	1.382 (3)
C5—C6	1.387 (3)	C22—H22	0.9300
C5—H5	0.9300	C23—C24	1.365 (4)
C6—C7	1.459 (3)	C23—H23	0.9300
C7—N1	1.309 (3)	C24—C25	1.376 (4)
C7—C14	1.423 (3)	C24—H24	0.9300
C8—N1	1.375 (3)	C25—C26	1.388 (3)
C8—C9	1.410 (3)	C25—H25	0.9300
C8—C13	1.416 (3)	C26—O1	1.376 (2)
C9—C10	1.362 (3)	C27—O1	1.433 (2)
C9—H9	0.9300	C27—C28	1.500 (3)
C10—C11	1.395 (3)	C27—H27	0.9800
C10—H10	0.9300	C28—C33	1.384 (3)
C11—C12	1.365 (3)	C28—C29	1.389 (3)
C11—H11	0.9300	C29—C30	1.376 (3)
C12—C13	1.407 (3)	C29—H29	0.9300
C12—H12	0.9300	C30—C31	1.374 (3)
C13—N2	1.383 (2)	C30—H30	0.9300
C14—N2	1.301 (2)	C31—C32	1.371 (3)
C14—C15	1.534 (3)	C31—Cl1	1.744 (2)
C15—N3	1.482 (2)	C32—C33	1.380 (3)
C15—C20	1.574 (2)	C32—H32	0.9300
C16—N3	1.436 (3)	C33—H33	0.9300
C16—S1	1.855 (2)	N4—O2	1.213 (2)
C16—H16A	0.9700	N4—O3	1.213 (2)
C16—H16B	0.9700	C34—Cl4	1.702 (4)
C17—C18	1.542 (3)	C34—Cl3	1.713 (5)
C17—S1	1.806 (2)	C34—Cl2'	1.716 (4)
C17—H17A	0.9700	C34—Cl2	1.740 (4)
C17—H17B	0.9700	C34—Cl3'	1.741 (5)
C18—N3	1.459 (2)	C34—Cl4'	1.789 (5)
C18—C19	1.529 (3)	C34—H34	0.9800
			
C2—C1—C6	119.32 (18)	C19—C20—H20	108.0
C2—C1—C15	129.22 (18)	C15—C20—H20	108.0
C6—C1—C15	111.44 (17)	C26—C21—C22	118.26 (19)
C1—C2—C3	119.0 (2)	C26—C21—C20	120.69 (18)
C1—C2—H2	120.5	C22—C21—C20	120.98 (19)
C3—C2—H2	120.5	C23—C22—C21	120.6 (2)
C4—C3—C2	121.1 (2)	C23—C22—H22	119.7
C4—C3—H3	119.4	C21—C22—H22	119.7
C2—C3—H3	119.4	C24—C23—C22	120.2 (2)
C5—C4—C3	120.6 (2)	C24—C23—H23	119.9
C5—C4—H4	119.7	C22—C23—H23	119.9
C3—C4—H4	119.7	C23—C24—C25	120.7 (2)
C4—C5—C6	118.6 (2)	C23—C24—H24	119.7
C4—C5—H5	120.7	C25—C24—H24	119.7
C6—C5—H5	120.7	C24—C25—C26	119.2 (2)
C5—C6—C1	121.27 (19)	C24—C25—H25	120.4
C5—C6—C7	129.75 (19)	C26—C25—H25	120.4
C1—C6—C7	108.95 (16)	O1—C26—C21	122.08 (18)
N1—C7—C14	123.79 (18)	O1—C26—C25	116.8 (2)
N1—C7—C6	128.09 (17)	C21—C26—C25	121.1 (2)
C14—C7—C6	108.01 (16)	O1—C27—C28	108.27 (15)
N1—C8—C9	119.51 (18)	O1—C27—C19	108.82 (15)
N1—C8—C13	121.63 (17)	C28—C27—C19	117.68 (16)
C9—C8—C13	118.84 (19)	O1—C27—H27	107.2
C10—C9—C8	120.1 (2)	C28—C27—H27	107.2
C10—C9—H9	120.0	C19—C27—H27	107.2
C8—C9—H9	120.0	C33—C28—C29	118.66 (18)
C9—C10—C11	121.0 (2)	C33—C28—C27	122.61 (18)
C9—C10—H10	119.5	C29—C28—C27	118.72 (17)
C11—C10—H10	119.5	C30—C29—C28	120.79 (18)
C12—C11—C10	120.6 (2)	C30—C29—H29	119.6
C12—C11—H11	119.7	C28—C29—H29	119.6
C10—C11—H11	119.7	C31—C30—C29	119.2 (2)
C11—C12—C13	119.9 (2)	C31—C30—H30	120.4
C11—C12—H12	120.0	C29—C30—H30	120.4
C13—C12—H12	120.0	C32—C31—C30	121.22 (19)
N2—C13—C12	118.86 (18)	C32—C31—Cl1	120.13 (17)
N2—C13—C8	121.60 (18)	C30—C31—Cl1	118.63 (17)
C12—C13—C8	119.54 (18)	C31—C32—C33	119.29 (19)
N2—C14—C7	123.09 (17)	C31—C32—H32	120.4
N2—C14—C15	126.01 (16)	C33—C32—H32	120.4
C7—C14—C15	110.84 (16)	C32—C33—C28	120.76 (19)
N3—C15—C14	110.17 (14)	C32—C33—H33	119.6
N3—C15—C1	118.11 (16)	C28—C33—H33	119.6
C14—C15—C1	100.26 (14)	C7—N1—C8	114.63 (16)
N3—C15—C20	103.63 (14)	C14—N2—C13	114.98 (16)
C14—C15—C20	114.11 (15)	C16—N3—C18	110.27 (15)
C1—C15—C20	111.03 (14)	C16—N3—C15	120.50 (15)
N3—C16—S1	108.00 (14)	C18—N3—C15	112.00 (14)
N3—C16—H16A	110.1	O2—N4—O3	124.58 (18)
S1—C16—H16A	110.1	O2—N4—C19	118.49 (17)
N3—C16—H16B	110.1	O3—N4—C19	116.88 (18)
S1—C16—H16B	110.1	C26—O1—C27	114.72 (15)
H16A—C16—H16B	108.4	C17—S1—C16	93.00 (10)
C18—C17—S1	104.77 (14)	Cl4—C34—Cl3	120.1 (3)
C18—C17—H17A	110.8	Cl4—C34—Cl2'	109.5 (3)
S1—C17—H17A	110.8	Cl3—C34—Cl2'	117.4 (4)
C18—C17—H17B	110.8	Cl4—C34—Cl2	116.2 (3)
S1—C17—H17B	110.8	Cl3—C34—Cl2	108.3 (4)
H17A—C17—H17B	108.9	Cl2'—C34—Cl2	9.0 (5)
N3—C18—C19	101.95 (14)	Cl4—C34—Cl3'	111.2 (4)
N3—C18—C17	108.61 (16)	Cl3—C34—Cl3'	15.4 (5)
C19—C18—C17	116.08 (17)	Cl2'—C34—Cl3'	113.5 (4)
N3—C18—H18	110.0	Cl2—C34—Cl3'	104.8 (4)
C19—C18—H18	110.0	Cl4—C34—Cl4'	20.5 (6)
C17—C18—H18	110.0	Cl3—C34—Cl4'	107.1 (5)
N4—C19—C20	111.80 (15)	Cl2'—C34—Cl4'	106.6 (3)
N4—C19—C18	110.45 (15)	Cl2—C34—Cl4'	110.7 (4)
C20—C19—C18	104.34 (15)	Cl3'—C34—Cl4'	95.2 (8)
N4—C19—C27	107.53 (15)	Cl4—C34—H34	103.2
C20—C19—C27	108.23 (15)	Cl3—C34—H34	103.2
C18—C19—C27	114.53 (16)	Cl2'—C34—H34	100.0
C21—C20—C19	113.44 (16)	Cl2—C34—H34	103.2
C21—C20—C15	115.47 (15)	Cl3'—C34—H34	118.5
C19—C20—C15	103.65 (14)	Cl4'—C34—H34	123.3
C21—C20—H20	108.0		
			
C6—C1—C2—C3	1.6 (3)	C15—C20—C21—C22	70.2 (2)
C15—C1—C2—C3	−179.92 (19)	C26—C21—C22—C23	−0.2 (3)
C1—C2—C3—C4	−1.6 (3)	C20—C21—C22—C23	176.7 (2)
C2—C3—C4—C5	0.3 (3)	C21—C22—C23—C24	−1.2 (4)
C3—C4—C5—C6	0.9 (3)	C22—C23—C24—C25	1.3 (4)
C4—C5—C6—C1	−0.9 (3)	C23—C24—C25—C26	0.1 (4)
C4—C5—C6—C7	176.9 (2)	C22—C21—C26—O1	178.72 (18)
C2—C1—C6—C5	−0.4 (3)	C20—C21—C26—O1	1.8 (3)
C15—C1—C6—C5	−179.12 (18)	C22—C21—C26—C25	1.6 (3)
C2—C1—C6—C7	−178.52 (17)	C20—C21—C26—C25	−175.35 (18)
C15—C1—C6—C7	2.7 (2)	C24—C25—C26—O1	−178.82 (19)
C5—C6—C7—N1	0.5 (3)	C24—C25—C26—C21	−1.5 (3)
C1—C6—C7—N1	178.44 (19)	N4—C19—C27—O1	−58.19 (19)
C5—C6—C7—C14	−175.9 (2)	C20—C19—C27—O1	62.75 (18)
C1—C6—C7—C14	2.1 (2)	C18—C19—C27—O1	178.65 (14)
N1—C8—C9—C10	−175.5 (2)	N4—C19—C27—C28	65.4 (2)
C13—C8—C9—C10	2.8 (3)	C20—C19—C27—C28	−173.70 (15)
C8—C9—C10—C11	−0.3 (4)	C18—C19—C27—C28	−57.8 (2)
C9—C10—C11—C12	−1.9 (4)	O1—C27—C28—C33	28.0 (3)
C10—C11—C12—C13	1.5 (4)	C19—C27—C28—C33	−95.8 (2)
C11—C12—C13—N2	−178.8 (2)	O1—C27—C28—C29	−150.91 (18)
C11—C12—C13—C8	1.1 (3)	C19—C27—C28—C29	85.3 (2)
N1—C8—C13—N2	−5.0 (3)	C33—C28—C29—C30	2.1 (3)
C9—C8—C13—N2	176.71 (18)	C27—C28—C29—C30	−178.90 (19)
N1—C8—C13—C12	175.10 (19)	C28—C29—C30—C31	−0.7 (3)
C9—C8—C13—C12	−3.2 (3)	C29—C30—C31—C32	−1.1 (3)
N1—C7—C14—N2	−5.4 (3)	C29—C30—C31—Cl1	177.28 (17)
C6—C7—C14—N2	171.10 (17)	C30—C31—C32—C33	1.5 (3)
N1—C7—C14—C15	177.37 (17)	Cl1—C31—C32—C33	−176.85 (17)
C6—C7—C14—C15	−6.1 (2)	C31—C32—C33—C28	−0.1 (3)
N2—C14—C15—N3	−44.7 (2)	C29—C28—C33—C32	−1.7 (3)
C7—C14—C15—N3	132.38 (16)	C27—C28—C33—C32	179.35 (19)
N2—C14—C15—C1	−169.92 (17)	C14—C7—N1—C8	2.2 (3)
C7—C14—C15—C1	7.18 (18)	C6—C7—N1—C8	−173.56 (18)
N2—C14—C15—C20	71.4 (2)	C9—C8—N1—C7	−179.09 (19)
C7—C14—C15—C20	−111.55 (17)	C13—C8—N1—C7	2.7 (3)
C2—C1—C15—N3	55.9 (3)	C7—C14—N2—C13	2.9 (3)
C6—C1—C15—N3	−125.49 (17)	C15—C14—N2—C13	179.67 (16)
C2—C1—C15—C14	175.49 (19)	C12—C13—N2—C14	−178.15 (18)
C6—C1—C15—C14	−5.90 (19)	C8—C13—N2—C14	2.0 (3)
C2—C1—C15—C20	−63.6 (3)	S1—C16—N3—C18	28.37 (19)
C6—C1—C15—C20	115.06 (17)	S1—C16—N3—C15	−104.55 (16)
S1—C17—C18—N3	36.06 (19)	C19—C18—N3—C16	−165.78 (16)
S1—C17—C18—C19	150.14 (14)	C17—C18—N3—C16	−42.7 (2)
N3—C18—C19—N4	158.18 (15)	C19—C18—N3—C15	−28.66 (19)
C17—C18—C19—N4	40.4 (2)	C17—C18—N3—C15	94.39 (19)
N3—C18—C19—C20	37.89 (18)	C14—C15—N3—C16	−97.3 (2)
C17—C18—C19—C20	−79.93 (19)	C1—C15—N3—C16	16.9 (2)
N3—C18—C19—C27	−80.24 (18)	C20—C15—N3—C16	140.19 (17)
C17—C18—C19—C27	161.94 (16)	C14—C15—N3—C18	130.46 (16)
N4—C19—C20—C21	81.17 (19)	C1—C15—N3—C18	−115.26 (18)
C18—C19—C20—C21	−159.45 (15)	C20—C15—N3—C18	7.99 (19)
C27—C19—C20—C21	−37.1 (2)	C20—C19—N4—O2	−19.1 (3)
N4—C19—C20—C15	−152.85 (15)	C18—C19—N4—O2	−134.8 (2)
C18—C19—C20—C15	−33.46 (18)	C27—C19—N4—O2	99.6 (2)
C27—C19—C20—C15	88.90 (17)	C20—C19—N4—O3	163.49 (18)
N3—C15—C20—C21	140.83 (16)	C18—C19—N4—O3	47.8 (2)
C14—C15—C20—C21	21.0 (2)	C27—C19—N4—O3	−77.8 (2)
C1—C15—C20—C21	−91.4 (2)	C21—C26—O1—C27	25.0 (2)
N3—C15—C20—C19	16.14 (18)	C25—C26—O1—C27	−157.73 (18)
C14—C15—C20—C19	−103.68 (17)	C28—C27—O1—C26	173.69 (15)
C1—C15—C20—C19	143.92 (15)	C19—C27—O1—C26	−57.3 (2)
C19—C20—C21—C26	6.5 (2)	C18—C17—S1—C16	−16.90 (16)
C15—C20—C21—C26	−113.0 (2)	N3—C16—S1—C17	−5.54 (16)
C19—C20—C21—C22	−170.39 (17)		

### Hydrogen-bond geometry (Å, º)

**Table:**

*D*—H···*A*	*D*—H	H···*A*	*D*···*A*	*D*—H···*A*
C34—H34···N1	0.98	2.59	3.401 (3)	140
C27—H27···N2	0.98	2.27	3.200 (3)	158
