# Supplementary figures and images for: Crystal structure of 6-(4-chloro­phen­yl)-6a-nitro-6,6a,6b,7,9,11a-hexa­hydro­spiro[chromeno[3′,4′:3,4]pyrrolo­[1,2-c]thia­zole-11,11′-indeno­[1,2-b]quinoxaline] chloro­form monosolvate
Source: Acta Crystallogr Sect E Struct Rep Online. 2014 Sep 20;70(Pt 10):o1111–2. doi: 10.1107/S1600536814020601 (PMC4257185; doi:10.1107/S1600536814020601)

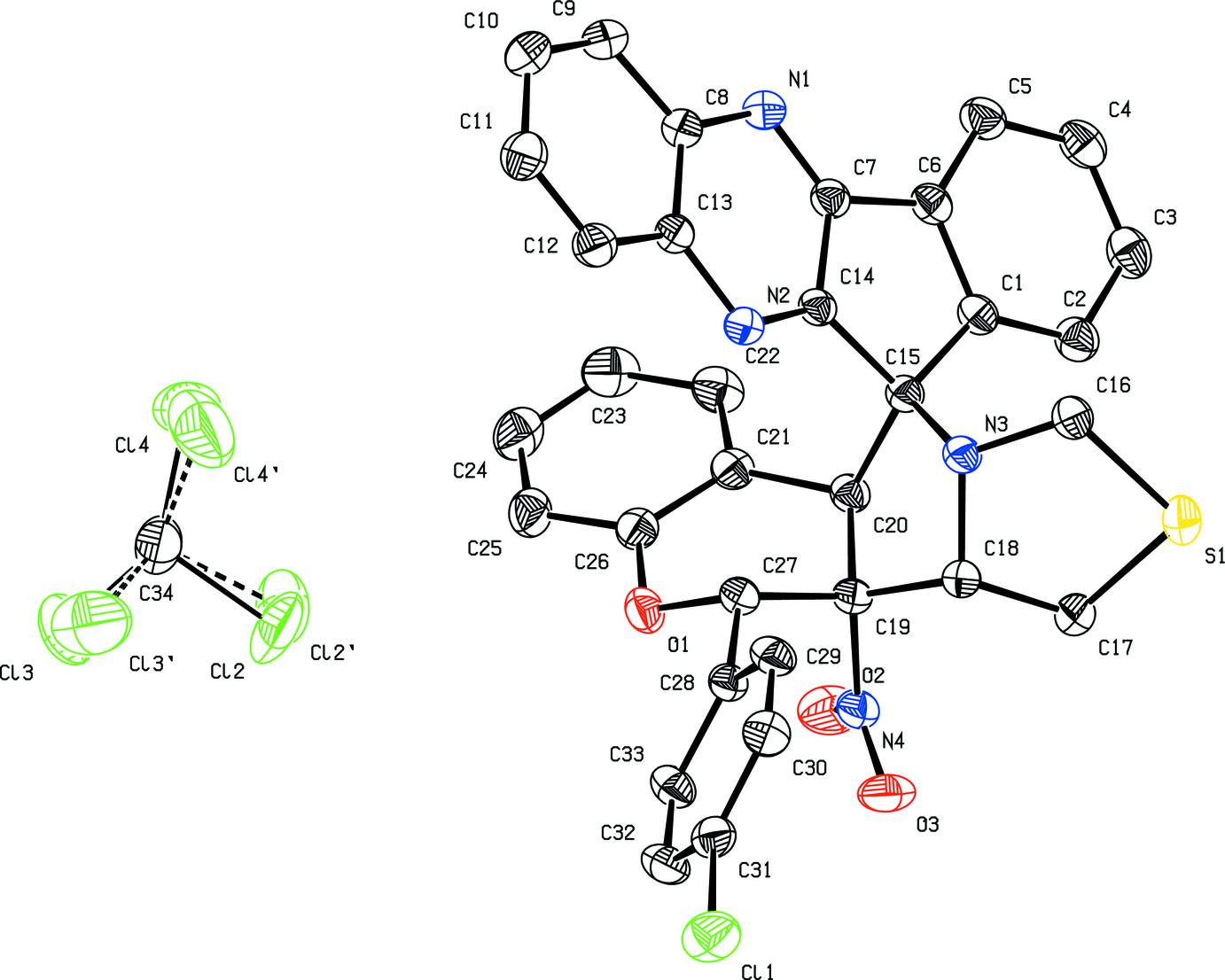

Supplement: Supplementary file 3 [file e-70-o1111-fig1.tif]

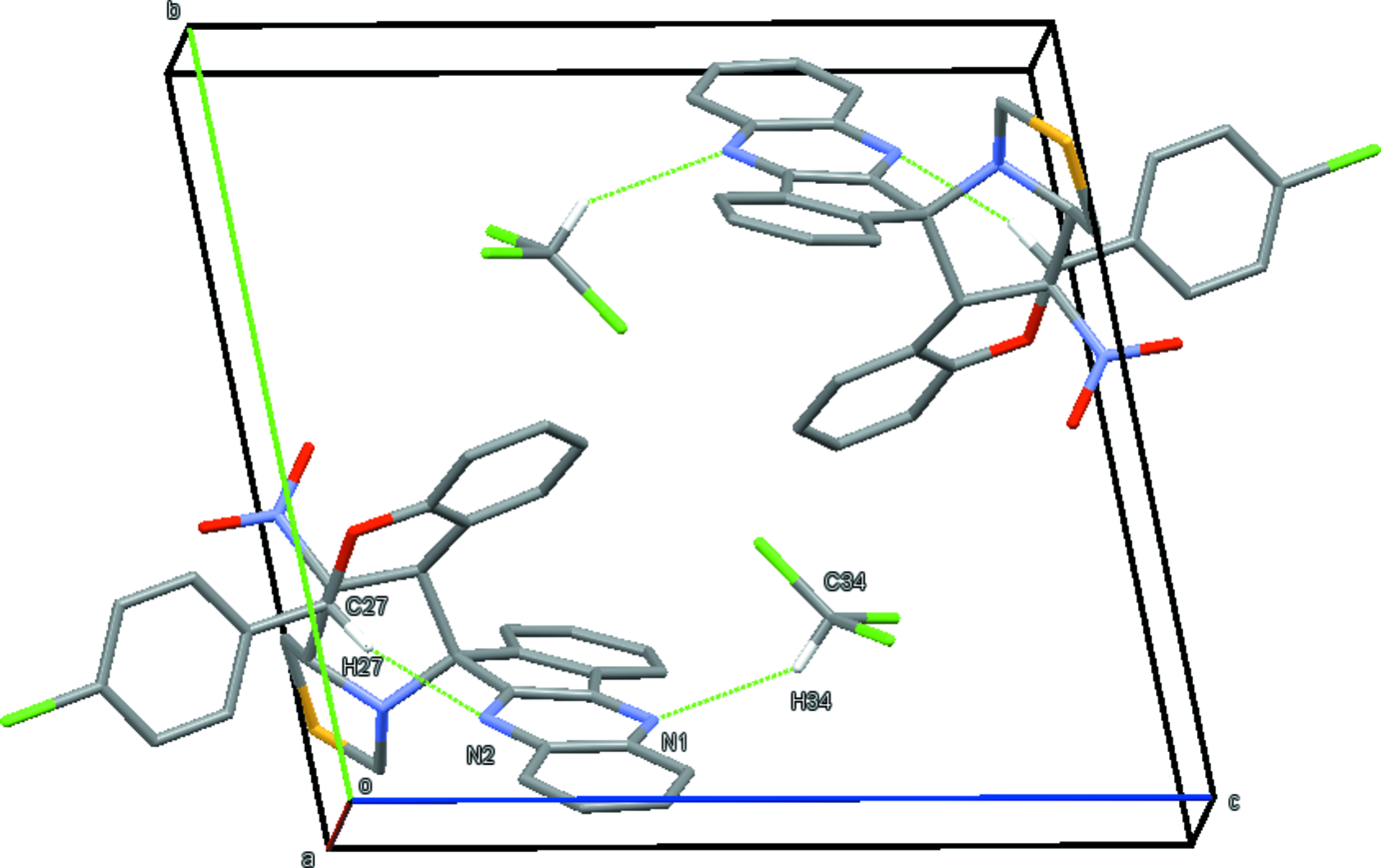

Supplement: Supplementary file 4 [file e-70-o1111-fig2.tif]
